# Initial experience with [^18^F]DPA-714 TSPO-PET to image inflammation in primary angiitis of the central nervous system

**DOI:** 10.1007/s00259-019-04662-4

**Published:** 2020-01-20

**Authors:** Philipp Backhaus, Wolfgang Roll, Carolin Beuker, Bastian Zinnhardt, Robert Seifert, Christian Wenning, Michel Eisenblätter, Christian Thomas, Antje Schmidt-Pogoda, Daniel Strunk, Stefan Wagner, Andreas Faust, Frank Tüttelmann, Albrecht Röpke, Andreas H. Jacobs, Walter Stummer, Heinz Wiendl, Sven G. Meuth, Michael Schäfers, Oliver Grauer, Jens Minnerup

**Affiliations:** 1 Department of Nuclear Medicine, University Hospital Münster, Münster, Germany; 2 European Institute for Molecular Imaging, University of Münster, Münster, Germany; 3 Department of Neurology with Institute of Translational Neurology, University Hospital Münster, Münster, Germany; 4 Institute of Clinical Radiology, University Hospital Münster, Münster, Germany; 5 Institute of Neuropathology, University Hospital Münster, Münster, Germany; 6 Institute of Human Genetics, University Hospital Münster, Münster, Germany; 7 Department of Geriatrics, Johanniter Hospital, Evangelische Kliniken, Bonn, Germany; 8 Department of Neurosurgery, University Hospital Münster, Münster, Germany

**Keywords:** Search items: DPA-714, TSPO, Vasculitis, PET-MRI, Microglia

## Abstract

**Purpose:**

Primary angiitis of the central nervous system (PACNS) is a heterogeneous, rare, and poorly understood inflammatory disease. We aimed at non-invasive imaging of activated microglia/macrophages in patients with PACNS by PET-MRI targeting the translocator protein (TSPO) with [^18^F]DPA-714 to potentially assist differential diagnosis, therapy monitoring, and biopsy planning.

**Methods:**

In total, nine patients with ischemic stroke and diagnosed or suspected PACNS underwent [^18^F]DPA-714-PET-MRI. Dynamic PET scanning was performed for 60 min after injection of 233 ± 19 MBq [^18^F]DPA-714, and MRI was simultaneously acquired.

**Results:**

In two PACNS patients, [^18^F]DPA-714 uptake patterns exceeded MRI correlates of infarction, whereas uptake was confined to the infarct in four patients where initial suspicion of PACNS could not be confirmed. About three patients with PACNS or cerebral predominant lymphocytic vasculitis showed no or only faintly increased uptake. Short-term [^18^F]DPA-714-PET follow-up in a patient with PACNS showed reduced lesional [^18^F]DPA-714 uptake after anti-inflammatory treatment. Biopsy in the same patient pinpointed the source of tracer uptake to TSPO-expressing immune cells.

**Conclusions:**

[^18^F]DPA-714-PET imaging may facilitate the diagnosis and treatment monitoring of PACNS. Further studies are needed to fully understand the potential of TSPO-PET in deciphering the heterogeneity of the disease.

**Electronic supplementary material:**

The online version of this article (10.1007/s00259-019-04662-4) contains supplementary material, which is available to authorized users.

## Introduction

Primary angiitis of the central nervous system (PACNS) represents a rare, severe, and poorly understood inflammatory disease affecting vessels of the central nervous system (CNS) [1]. The disease is characterized by transmural inflammation with damage to the vascular wall [2, 3]. Due to the rareness of the disease (estimated prevalence of 2.4/million person-years in North America [4]), its non-specific clinical and imaging manifestations, and a broad spectrum of differential diagnoses, PACNS is a clinical challenge. Current diagnostic criteria were implemented by Calabrese and Mallek in 1988 [5]. Accordingly, definite PACNS can only be confirmed by invasive brain biopsies proofing inflammation of cerebral vessels. However, due to the focal and segmental distribution of the disease, only 50 to 75% of the brain biopsies are positive [6, 7]. Probable PACNS is rendered without histological verification but with positive findings on angiogram and cerebrospinal fluid (CSF) analysis consistent with PACNS (i.e., mild lympho-monocytic pleocytosis or protein elevation, occasionally presence of oligoclonal bands) and abnormal MRI. Suspicious findings on MRI include multifocal ischemic and hemorrhagic lesions, vessel wall contrast enhancement using blood suppression techniques [8], leptomeningeal enhancement, and vessel caliber abnormalities in MR angiography [9]. Although MRI often raises primary suspicion of PACNS, its findings are not specific on their own. Both vessel wall caliber abnormalities and vessel wall enhancement are commonly encountered in several other vascular disorders, e.g., atherosclerosis and radiation vasculopathy [10–13]. Repeated neurological examinations and periodic MRI and MR angiography during and after therapy are recommended for assessing disease activity [3]. A more specific and sensitive imaging biomarker of vessel wall and CNS inflammation would be highly desirable for primary diagnosis and follow-up of PACNS.

The inflammatory infiltrate in PACNS typically shows a granulomatous, lymphocytic, or necrotizing pattern [4]. Furthermore, the inflammatory response involves microglial/macrophage activation and infiltration [14–16]. FDG-PET can assess elevated glycolysis of inflammatory cells and is a promising imaging biomarker of vessel wall inflammation in large-vessel vasculitis in the body [17]. However, the high physiologic FDG uptake of the CNS complicates imaging of intracranial vasculitis as it can mask uptake of adjacent vessels. Similarly, increased inflammation in CNS tissue due too small vessel vasculitis or ischemia is not necessarily accompanied by increased FDG uptake [18]. These limitations of FDG-PET in intracranial vascular disease demand novel imaging biomarkers. The 18-kDa translocator protein (TSPO), also known as peripheral benzodiazepine receptor (PBR), is a mitochondrial transmembrane protein expressed on the mitochondrial membrane of activated microglia cells and infiltrating macrophages [19]. Following inflammatory triggers, its expression is upregulated, and it is considered a sensitive marker for neuroinflammation [19–21].

In this case series, we reported nine patients with acute stroke symptoms and diagnosed or suspected PACNS where we visualized TSPO expression with [^18^F]DPA-714-PET-MRI. The aim of our study was to assess the potential of TSPO-PET-MRI to assist in differential diagnosis, therapy monitoring, and biopsy planning in suspected PACNS. Immunohistochemistry of patient biopsy samples was performed to correlate microscopic patterns of cellular inflammation and TSPO expression with the observed macroscopic [^18^F]DPA-714-PET uptake pattern in individual patients.

## Materials and methods

### Patients

A total of nine patients underwent [^18^F]DPA-714-PET-MRI (Siemens Biograph mMR, 3 Tesla) at the Department of Nuclear Medicine, University of Münster, Germany, in the frame of compassionate use (“individueller Heilversuch”). All patients were hospitalized in the Department of Neurology, University of Münster, Germany, for work-up of stroke with either diagnosed or suspected PACNS at the time point of PET. Table 1 gives further details on the patients’ characteristics. In one of the PACNS patients, follow-up imaging was performed after initiation of anti-inflammatory treatment. In two patients (patient #1, with vasculitic ischemia; patient #9, with non-vasculitic ischemia) histological staining of biopsy samples of [^18^F]DPA-714-positive tissue was performed. Numbering of patients in the manuscript is not chronological but ordered by final diagnosis/appearance in the manuscript.

**Table 1:** Patient characteristics

#	Sex & age	Diagnosis	Biopsy	MRI findings	TSPO-PET	Immunosuppressive therapy & onset before scan	Genetic binding type
1	M−50	PACNS (diagnosis established by PET/MRI targeted biopsy)	Perivascular necrosis, accumulated TSPO-positive microglia/macrophages	Sub-centimeter lesions with contrast medium leakage in the left hemisphere and unilateral atrophy of the left cerebral peduncle and left brain stem	Tracer uptake in left-sided basal ganglia, cerebral peduncle and left brain stem	None	Medium affinity
2	F–60	PACNS (diagnosis established by angiography before PET scan)	–	Subacute infarctions of the right splenium and right occipital pole. Right ICA occlusion and left ICA stenosis, both with vessel wall enhancement	Right-dominant tracer uptake of the whole cerebral hemisphere	Glucocorticoids 4 days	Medium affinity
3	F–45	PACNS (diagnosis established by angiography shortly before PET scan)	–	Infarction of the left internal capsule and right cerebellum	No pathologic tracer uptake	Glucocorticoids 9 days, cyclophosphamide 4 days	Medium affinity
4	M–52	PACNS (diagnosis established by angiography shortly before scan)	–	Small subacute stroke semi oval center, ubiquitous artery inflammation	No pathologic tracer uptake	Glucocorticoids 13 days	Medium affinity
5	F–37	Cerebral predominant lymphocytic vasculitis (diagnosis established by biopsy before scan)	Perivascular lymphocytic infiltrates (muscle and skin-biopsy)	Several acute and subacute infarctions at both hemispheres	Mild tracer uptake in the peri-infarct region of subacute infarction	Glucocorticoids 4 days	Not sequenced
6	M–24	Singular media infarct of unknown etiology	–	Infarction of the right MCA territory (corona radiata, temporo-occipital and insular)	Focal tracer uptake at the infarction site	Glucocorticoids 4 days	High affinity
7	M–51	Singular media infarct due to arteriosclerotic large vessel disease	–	Infarction of the right MCA territory	Strong tracer uptake in the peri-infarct regions	None	Not sequenced
8	M–71	Several subacute partially hemorrhagic infarctions of unknown etiology	–	Subacute partially hemorrhagic infarctions temporal right and occipital left. New diffusion restrictions occipital right and left splenium	Mild tracer uptake in the peri-infarct regions of subacute infarctions	None	High affinity
9	M–32	Moyamoya disease (diagnosis established by angiography and biopsy)	No perivascular inflammation. Mildly elevated diffuse TSPO-expression	Bilateral infarction of the ACA territories	Focal tracer uptake at the infarction sites	Glucocorticoids 7 days	High affinity

Retrospective analysis of imaging data, biopsy material, and clinical information were conducted with the ethical standards of the institutional research committee (*Ethikkommission der Ärztekammer Westfalen Lippe*; reference number 2019–591-f-S) and with the principles of the 1964 Declaration of Helsinki and its later amendments or comparable ethical standards. Patients gave written informed consent on both the examination and the genotyping of the Ala147Thr-polymorphism to characterize individual TSPO affinity. All patients gave written consent on anonymous case report publication.

### Preparation of [^18^F]DPA-714

[^18^F]DPA-714 was prepared in a GE TRACERlab FX-FN synthesizer in accordance with a published procedure [22]. In brief, after the one-step synthesis consisting of a nucleophilic substitution of the precursor *N,N*-diethyl-2-(2-(4-(2-toluenesulfonyloxyethoxy)-phenyl)5,7dimethylpyrazolo[1,5a] pyrimidin-3-yl) acetamid with [^18^F]fluoride, the crude product mixture was purified by high-performance liquid chromatography (λ, 254 nm; flow, 3.0 mL/min; column, ACE 126–2510, 10 mm × 250 mm; eluent, 0.1 M NH_4_OAc/EtOH 6/4 (v/v) pH 10.0).The resulting product batch was subjected to a sterile filtration using a 0.2-μm filter (Sterifix® Paed, B. Braun) and dispensed automatically in a closed vial filling in 10-mL vials. The batch was released for human application after quality control (QC) including the determination of the pH value (4.0–8.5), the osmolality (< 3000 mOsm/kg), the radiochemical purity (≥ 95%), the filter integrity (“bubble-point” test), endotoxins (≤ 17.5 EU/mL), and the content of residual solvents and chemical impurities (DMSO, acetonitrile, EtOH, kryptofix® 222).

### PET-MRI protocol

Patients were injected intravenously with 233 ± 19 MBq [^18^F]DPA-714. After positioning of the head in the field-of-view of the PET-MRI, a dynamic PET scanning for 60 min was initiated with injection of the tracer. PET images were co-registered to simultaneously acquired, routine non-contrast enhanced MR images (axial FLAIR, SWI, DTI, and T2 TSE 3 mm of the brainstem and TOF angiography of intracranial arteries).

### Image analysis

Co-registered images were analyzed using syngo.via (version VB20A; Siemens Healthineers). Dynamic studies were analyzed using the in-house developed software tool, MEDgical. Atlas-based analysis of PET and MRI/DTI datasets was conducted with FMRIB library (FSL Version 5.0) [23, 24] using the ICBM-DTI-81 white-matter label atlas. Calculation of fractional anisotropy was performed based on DTI measurements using FreeSurfer. All scans were analyzed by board-certified nuclear medicine physicians and radiologists and reported in consensus. Lesion-to-contrast-ratios were assessed comparing a representative volume-of-interest (VOI) of the pathological uptake pattern to contralateral healthy tissue 30–60 min p.i. [25].

### Genetic analysis to characterize individual TSPO ligand affinity

The single-nucleotide polymorphism (SNP) c.439A > G (rs6971, p.Thr147Ala) was analyzed by direct Sanger sequencing. Genomic DNA was extracted from EDTA-preserved blood using standard techniques. PCR was carried out in a volume of 20 μl with approximately 200 ng DNA and 5 pmol/μl forward primer (5’-TCAGGTGGCATGACTGTTCC-3′) and reverse primer (5’-GCATGCAGAAAGCACAGGAC-3′) using Biotaq DNA polymerase and dNTPs (Bioline, Luckenwalde; Germany). For sequencing, the PCR products were treated with ExoSAP-IT (USB Corporation, Cleveland, OH, USA). The sequencing reaction was carried out using the BigDye Terminator v3.1 Cycle Sequencing Kit (Applied Biosystems, Carlsbad, CA, USA) and analyzed on a 3730 DNA Analyzer (Applied Biosystems).

### Histology and immunohistochemistry

Immunohistochemical staining of formalin-fixed and paraffin-embedded tissue for CD3 (mouse monoclonal, 1:25, Dako, Glostrup, Denmark), CD20 (mouse monoclonal, 1:700, Dako, Glostrup, Denmark), CD45 (mouse monoclonal, 1:800, Dako, Glostrup, Denmark), GFAP (rabbit polyclonal, 1:4000, Dako, Glostrup, Denmark), CD68 (supernatant from KiM1P hybridoma cells, kindly provided by Prof. Klapper, Institute of Pathology, Kiel, 1:5000), and TSPO (rabbit monoclonal, 1:400, Abcam, Cambridge, United Kingdom) was performed using the streptavidin-biotin method on an automated staining system (LINK48, DAKO). For double immunofluorescence (see Fig. 2b), slices were incubated as described previously [26, 27] with antibodies against TSPO (1:250, rabbit anti-TSPO, NBP1–95674, AB_11015478, Novus Biologicals, Cambridge, UK), Iba1 (1:250, rabbit anti α Iba1, 019–19,742, Wako Chemicals, Neuss, Germany), and GFAP (1:1000, chicken anti GFAP, ab4674). Alexa Fluor 488/555 (1:800; Life Technologies) and DSB-X™ Biotin Goat Anti-Chicken IgG (1:800; Life Technologies) were used as secondary antibodies. Double immunofluorescence for TSPO and Iba-1 was performed using a preconjugated TSPO antibody (1:100; anti-PBR antibody [EPR5384] (Alexa Fluor® 647) (ab199836)).

## Results

### Patient characteristics

Overall, nine patients (mean age, 46.9 years; range 24–71, 6 men) were examined with [^18^F]DPA-714-PET-MRI (Table 1). All patients were hospitalized because of acute stroke symptoms with diagnosed or suspected PACNS. Four patients were finally diagnosed with PACNS (patients #1–#4), one patient with cerebral manifestation of systemic vasculitis (patient #5), three patients with ischemic stroke of non-inflammatory origin (patients #6–#8), and one patient with moyamoya disease (patient #9). In a patient with PACNS (patient #1), definite diagnosis was confirmed by brain biopsy; in the remaining three patients (patients #2, #3, and #4), diagnosis of PACNS was based on characteristic patterns in angiography and clinical features. Diagnosis of systemic vasculitis in patient #5 was established by skin and muscle biopsies.

### TSPO imaging

Two patients with PACNS (patients #1 and #2) demonstrated regionally increased [^18^F]DPA-714 uptake that extended the area of infarction-related MRI abnormalities.

#### Patient #1

**Fig. 1:**
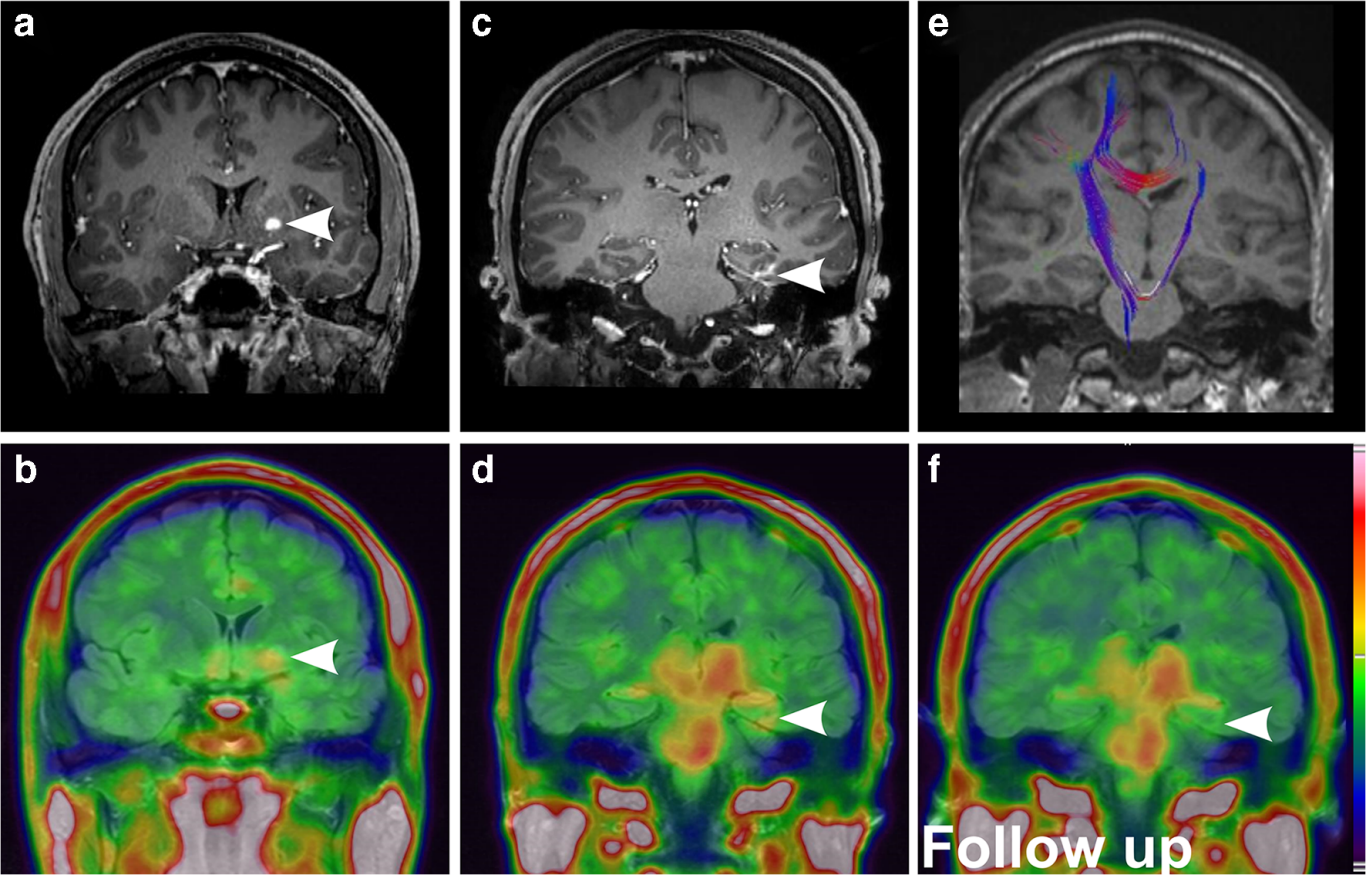
Imaging findings of patient #1 diagnosed with PACNS. Coronal T1wi after Gadovist® injection demonstrates the two major contrast enhancing lesion in the left putamen and left temporal lobe (**a, c,** arrows, contrast-enhanced MRI 2 weeks before PET scan). Coronal FLAIR/PET overlay demonstrates [^18^F]DPA-714 uptake of the lesions (**b, d**) with additional regional tracer uptake of the left corticospinal tract. (**e**) Tractography images overlaid with T1wi demonstrate reduced left sided fiber integrity. Follow-up FLAIR/PET, 4 weeks after induction of anti-inflammatory therapy, (**f**) demonstrates normalization of uptake in the left temporal lesion and constant uptake of the left corticospinal tract

**Fig. 2:**
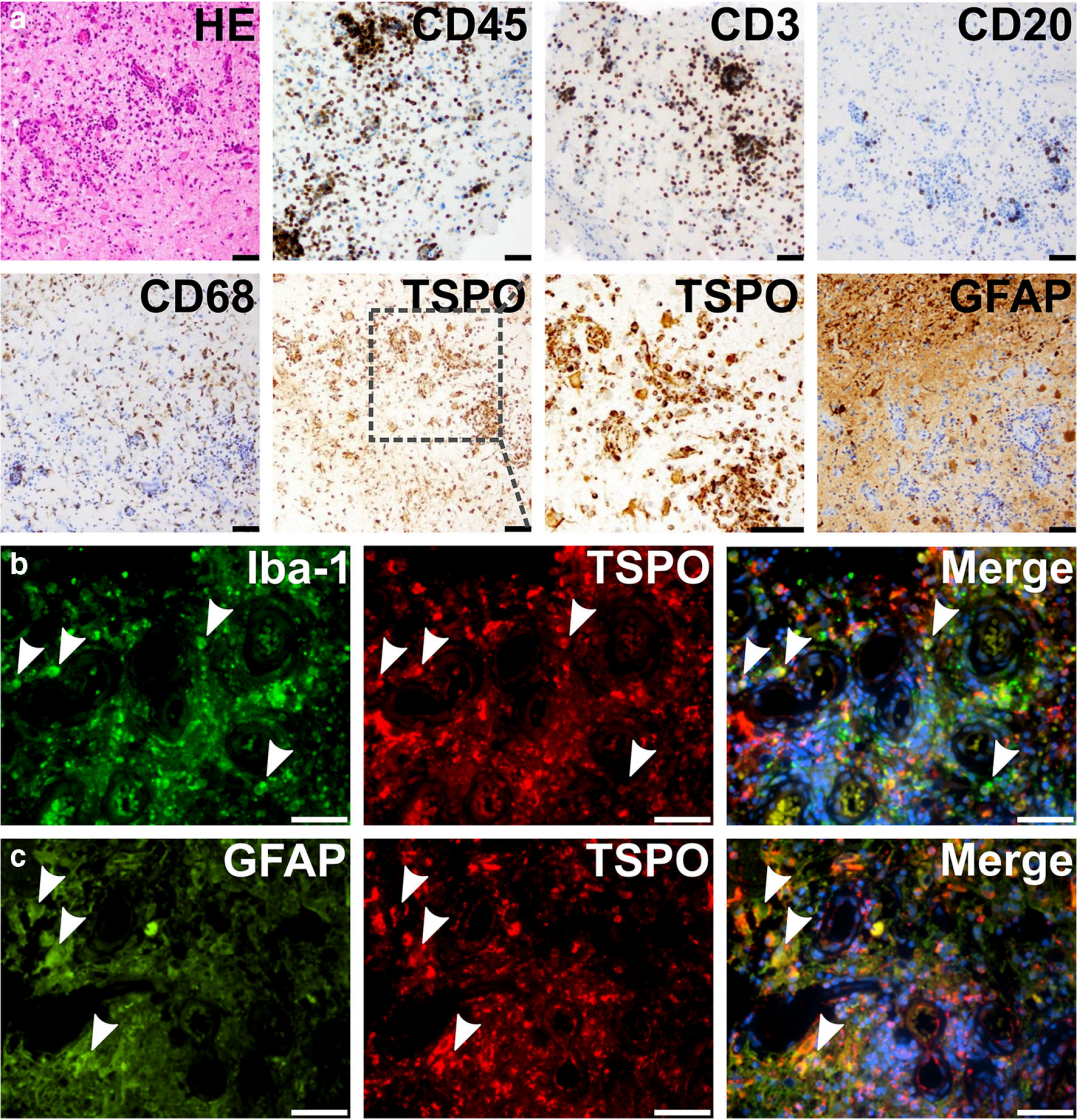
Histology of patient #1. (**a**) On histopathological examination brain tissue with reactive changes, small areas of necrosis and non-granulomatous inflammatory infiltrates were encountered. Inflammatory infiltrates were associated with intraparenchymal blood vessels and mainly comprise CD45- and CD3-positive T-lymphocytes with some intermingled CD20-positive B-cells and CD68-positive microglial cells. Of note, immune infiltrates strongly stain for TSPO. GFAP-staining shows reactive gliosis. (**b, c**) Immunohistochemistry indicates extensive TSPO expression in microglia/macrophages (**b**, Iba-1, arrows) and astrocytes (**c**, GFAP). All scale bars represent 50 μm

A 50-year-old male patient was hospitalized with right-sided slowly progressive spastic hemiplegia, hyperreflexia, and pyramidal tract signs. MRI demonstrated multiple small contrast-enhancing lesions of the left-sided basal ganglia, internal capsule, and basal temporal cortex. [^18^F]DPA-714-PET-MRI was performed to support the diagnostic work-up and to identify a representative lesion for brain biopsy. PET revealed increased focal uptake in the major lesion in the left putamen (SUV_max_ lesion-to-contralateral ratio (L/C): 1.3, Fig. 1 a, b) and the left temporal lobe (SUV_max_ L/C: 1.3, Fig. 1 c, d). Additionally, regional asymmetric uptake of [^18^F]DPA-714 was present within the left corticospinal tract including thalamus, internal capsule, cerebral peduncle (SUV_max_ L/C: 1.2), and brain stem (Fig. 1 d)**.** Here, the higher uptake was not confined to MR contrast enhancement but was instead accompanied by unilateral atrophy consistent with reduced fractional anisotropy (cerebral peduncle fractional anisotropy L/C: 0.75) as calculated from DTI (Fig. 1 e). Biopsy of the lesion in the left putamen revealed prominent perivascular T-lymphocytic inflammatory infiltrates, necroses as well as astro- and microgliosis (Fig. 2 a). In consideration of the results from histology, conventional imaging studies, and clinical appearance, PACNS was established as the diagnosis. Immunohistochemical staining for TSPO-positive cells, Iba1-positive microglia/macrophages, and GFAP-positive astrocytes additionally indicated extensive TSPO expression in activated microglia/macrophages and astrocytes (Fig. 2 b, c).

We performed a follow-up [^18^F]DPA-714 scan in patient #1 4 weeks after anti-inflammatory treatment initiation with high-dose intravenous corticosteroids to examine the changes in inflammation and immune cell infiltration. In this scan, pathologic [^18^F]DPA − 714 uptake in the temporal lesion was no longer visible (SUV_max_ L/C: 1.05 vs 1.3 at baseline), whereas the initial regional uptake of the left corticospinal tract remained unaltered (SUV_max_ L/C: 1.2 vs 1.2 at baseline, Fig. 1 f).

#### Patient #2

**Fig. 3:**
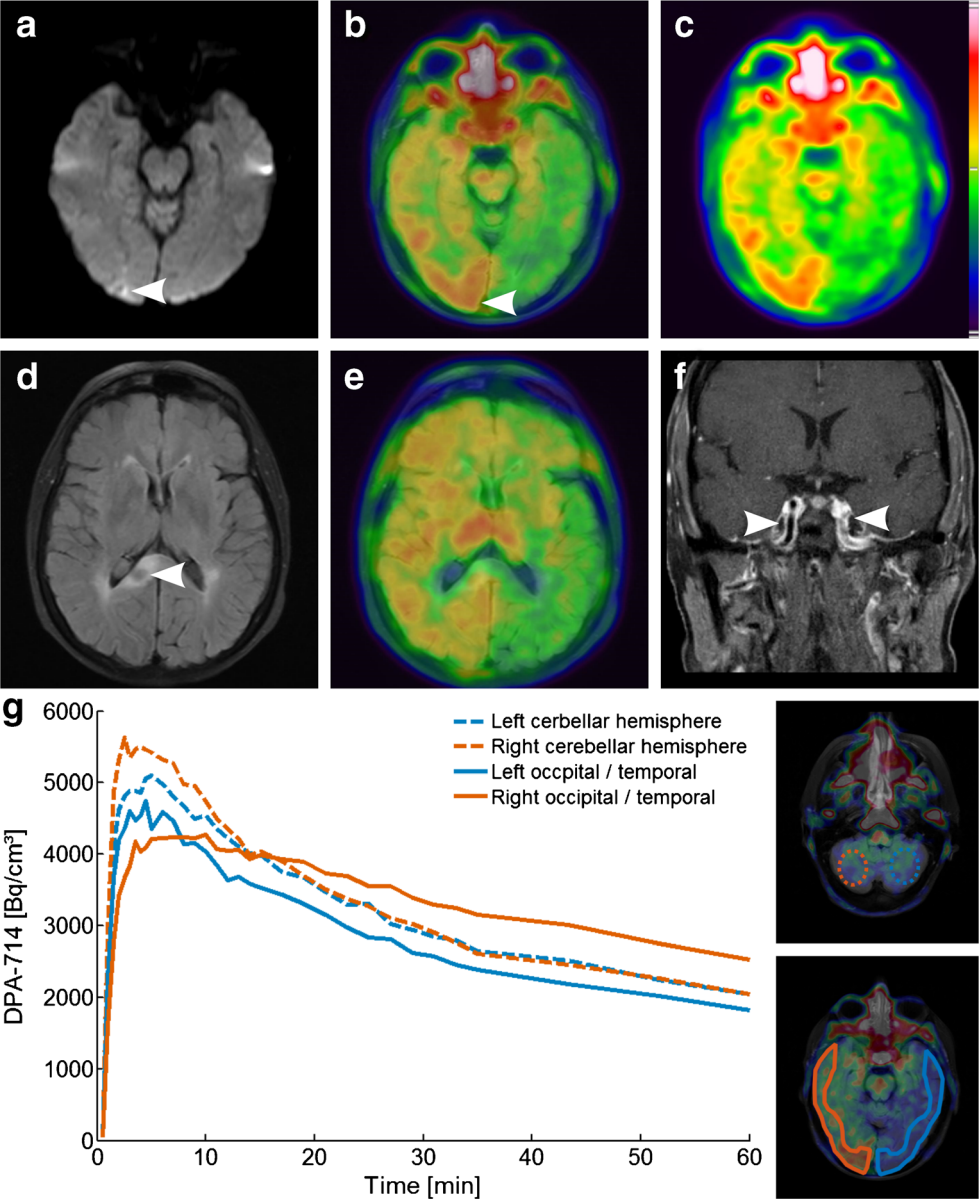
Imaging findings of patient #2 diagnosed with PACNS. [^18^F]DPA-714-PET images fused to FLAIR demonstrate elevated tracer uptake of the whole cerebral hemisphere. Right-sided occipital pole hotspot (**b**, arrow FLAIR/PET overlay, **c**, PET) corresponds to diffusion restriction as indicated by DTI (**a**, white arrow, MR scan 5 days before PET/MRI). FLAIR image of subacute ischemia in the right splenium (**d**, white arrow) corresponding to fusion image (**e**). Coronal T_1_wi after contrast agent application demonstrates vessel wall uptake (**f**, white arrows, contrast-enhanced MR scan 5 days before PET/MRI). (**g**) Time activity curves of symmetric ROIs on the occipital / temporal cortices and symmetrical spherical VOIs on the cerebellar hemispheres as indicated in the inset images

A 60-year-old female patient was hospitalized with disorientation and declined psychomotor performance indicating relapse of previously diagnosed PACNS. PET-MRI 4 days after corticosteroid therapy initiation showed a widespread increased cerebral uptake of [^18^F]DPA-714 in the whole right cerebral hemisphere (Fig. 3). In this case, tracer influx varied between the cerebral and cerebellar hemispheres as a result of the heavily impaired perfusion due to large arterial vessel involvement (Fig. 3 f, g)**.** Tracer uptake of the two cerebellar hemispheres converged over time; however, tracer uptake in the cerebral hemispheres continuously diverged with higher uptake in the right hemisphere putatively reflecting increased TSPO binding. Tracer uptake was especially elevated at the right occipital pole where diffusion restriction was apparent in DWI and ADC mapping indicating subacute ischemia (SUV_mean_ L/C: 1.6, Fig. 3 a-c). Another subacute infarction at the right splenium with extensive edema (Fig. 3 d, e) did not show particularly increased tracer accumulation compared to the overall elevated uptake of the right hemisphere (SUV_mean_ L/C: 1.2).

**Fig. 4:**
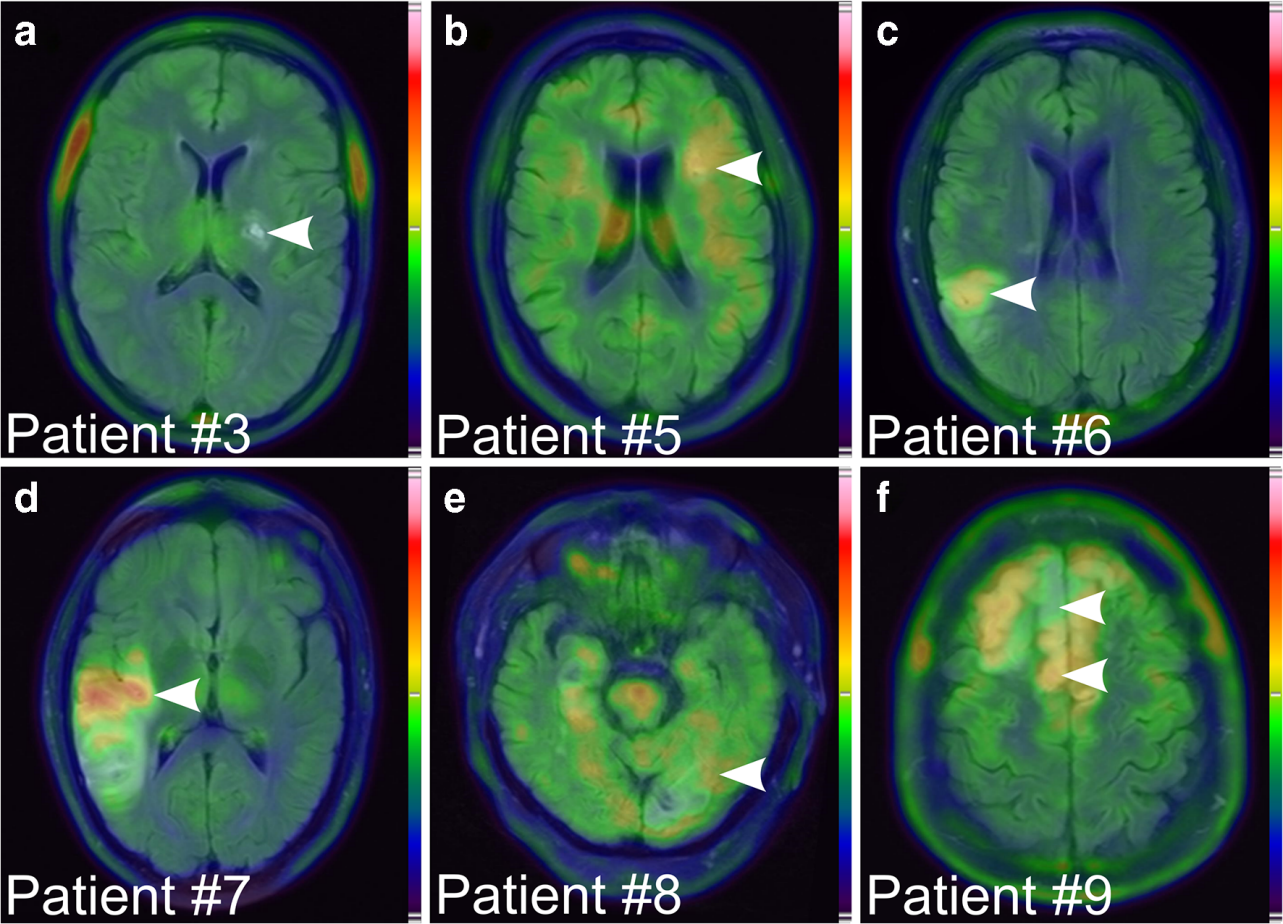
FLAIR/PET overlay of patient #3 (PACNS), #5 (lymphocytic vasculitis), #6, #7, #8 (stroke of non-vasculitis etiology), and #9 (moyamoya) (**a–f**, respectively). Arrows highlight the dominant lesions

The remaining two patients with PACNS (patients #3 and #4) [^18^F]DPA-714-PET-MRI revealed neither cerebral nor perivascular pathological tracer uptake patterns but multiple, [^18^F]DPA-714 negative, FLAIR-hyperintense subacute ischemic lesions (Fig. 4 a**,** Suppl. Fig. 1 a–f). Notably, these two patients had the longest interval between initiation of immunosuppressive therapy and PET scanning (9 and 13 days, respectively). Another patient with cerebral predominant lymphocytic vasculitis (patient # 5) demonstrated only faint uptake in the peri-infarct region of two subacute infarctions 4 days after the onset of immunosuppressive therapy (Fig. 4b**,** Suppl. Fig. 1 g–i).

Four patients were finally diagnosed with cerebral ischemia of non-vasculitis origin (patients #6–#9). All four patients showed tracer uptake with varying intensity at the ischemic and peri-infarct area not extending the MR correlates of ischemia (SUV_max_ L/C: patient #6: 1.4, #7: 2.0, #8: 1.1 and SUV_max_ frontal to parietal in patient #9: 1.4. Figure 4**c-f,** Suppl. Fig. 2). Whereas patients #6–#8 were diagnosed with stroke of arteriosclerotic or unknown origin, patient #9 was finally diagnosed with moyamoya disease based on biopsy results and invasive angiography. This patient demonstrated bifrontal uptake in wide overlap with FLAIR signal abnormalities (Fig. 4 f). Histological analysis of the biopsy sample of a [^18^F]DPA-714-positive frontal ischemic site revealed brain tissue with reactive changes and mild microgliosis. As compared to normal brain control tissue, intermingled microglial cells displayed TSPO expression (Suppl. Fig. 3)**.**

Pathological tracer uptake in the vascular wall of large vessels was not observed in any of the 9 patients, despite MRI-evident large-vessel involvement in 2 PACNS patients (#2 and #4) (e.g. Figure 3**f**).

## Discussion

Non-invasive imaging strategies to specifically visualize inflammation, the pathological hallmark of PACNS, are still missing. Only a few imaging studies report on inflammation imaging in (cerebral) vasculitis [28]. For instance, in patients with large-vessel vasculitis, the detection of vascular inflammatory activity has been demonstrated with the first-generation TSPO-PET-Tracer [^11^C]-PK11195 [29]. In a single case report, [^11^C](*R*)-PK11195 PET revealed an active intracranial inflammatory process with perivascular infiltrates and activated microglia [14]. We here demonstrate the feasibility of PET imaging with [^18^F]DPA-714, a second-generation PET-tracer with improved affinity and selectivity, to visualize inflammation-driven overexpression of TSPO in patients with PACNS. In our case series, two patients with PACNS showed widespread [^18^F]DPA-714 uptake exceeding MR correlates of ischemia. In contrast, uptake outside the ischemic area was not observed in any of our patients with stroke of non-vasculitis origin or moyamoya disease confirming preclinical and clinical imaging studies that TSPO is highly expressed by microglia/macrophages in the infarct core and peri-infarct areas in ischemic stroke with non-vasculitis origin [30–35].

The observed differences in increased uptake exceeding MRI correlates of ischemia in PACNS in contrast to uptake restricted to MR-abnormalities in stroke of non-inflammatory origin allow to raise the hypothesis of specific cerebral TSPO distribution in PACNS. In order to track the underlying cellular source, histological staining of biopsy samples of [^18^F]DPA-714-positive tissue was performed in two patients: One patient with vasculitic ischemia (patient #1) with an uptake pattern exceeding MRI changes and one patient with non-vasculitic ischemia (patient #9) with uptake confined to the MRI correlates of ischemia. The different pathophysiology in these two patients was indeed reflected by specific histologic patterns of TSPO-expressing cells, i.e., perivascular infiltrates in patient #1 and diffuse microgliosis reflecting postischemic inflammation in patient #9. These findings strengthen the hypothesis that the [^18^F]DPA-714 uptake pattern in patient #1 and #2 reflect ubiquitous perivascular inflammation of small vessels in contrast to the more confined uptake patterns resulting from postischemic inflammation. However, the specificity of the [^18^F]DPA-714 uptake in different locations remains a subject of discussion: In patient #1 Wallerian degeneration, the replacement of degenerated anterograde axons and myelin sheets by gliosis following proximal neuronal loss could have alternatively driven unilateral tracer accumulation in the left cerebral peduncle and brain stem. In contrast to the [^18^F]DPA-714 uptake foci in the left basal ganglia and temporal lobe, longitudinal areal uptake in cerebral brain stem, peduncle, and capsula interna was not entirely correlated with inflammatory MRI features. Instead, unilateral atrophy and reduced fractional anisotropy were observed in MRI – both potential correlates of axonal damage. TSPO-PET uptake in the process of Wallerian degeneration was previously observed in ischemic stroke patients [30]. Moreover, in contrast to focal TSPO uptake in lesions accompanied by blood-brain barrier leakage, regional uptake in the cerebral brain stem, peduncle, and capsula interna did not change following immunosuppressive treatment. This observation suggests a different cellular basis of TSPO expression between the two uptake patterns that likely reflect different etiologies. In patient #2 increased tracer uptake covered the whole right cerebral hemisphere, exceeding FLAIR signal alterations. Indeed, cases of unilateral PACNS have been described, which suggests that the uptake pattern may reflect small-vessel vasculitis [36]. Alternatively, MRI-occult (relative) ischemia due to the observed progressive right-sided carotid artery occlusion could have triggered the hemispheric tracer uptake as well. Notably, the patient demonstrated a fetal variant of the right posterior cerebral artery, which allows pinpointing the area of increased tracer uptake to the bed of the right internal carotid artery. Importantly, the discussed alternative pathophysiologic mechanisms in patient #1 and #2, i.e., Wallerian degeneration and MRI occult subtotal ischemia, are not specific features of PACNS but could also be recognized in stroke and vessel stenosis of non-vasculitis origin.

Besides the limited number of patients resulting from the rareness of PACNS, our study features further limitations: (1) Only patient #1, showing prominent [^18^F]DPA-714 uptake pattern, had PET-MRI before immunosuppressive treatment initiation. The other patients with vasculitis were examined 3–13 days after high-dose glucocorticoid therapy, and thus inflammation might already be suppressed hampering its visualization. In line with this hypothesis, follow-up TSPO-PET imaging in patient #1 (4 weeks after initiation of immunosuppressive therapy) showed a considerable decrease of lesional tracer uptake. Consequently, the long treatment interval of PACNS patients #3 and #4 (9 and 13 days after glucocorticoid treatment initiation, respectively) could explain the non-pathologic tracer uptake pattern. Early onset of immunosuppressive therapy is not only a specific limitation of our cohort but rather a general challenge in imaging PACNS as such an early intervention is the current clinical standard as soon as PACNS is suspected [37]. Thus, a certain delay for TSPO-PET imaging in the clinical routine is hard to overcome. (2) About three of five patients with vasculitis were diagnosed by angiography according to established criteria but not by biopsy; hence, differential diagnoses mimicking vasculitis are possible. (3) PACNS is a heterogeneous disease with different histopathological subtypes of inflammation, clinical presentations, and consequently imaging findings [4]. (4) A recently published comment by P. Zanotti-Fregonara and colleagues raised concerns on potential unspecific uptake of the TSPO tracer [^18^F]GE-180 due to (MRI occult) blood-brain barrier breakdown [38]. Although we utilized a radiotracer with different kinetic properties, unspecific uptake mechanisms have to be taken into account for [^18^F]DPA-714 as well. We did not acquire an arterial input function impeding conventional pharmacokinetic modeling. Healthy reference regions allow to perform pharmacokinetic modeling without an arterial input function, as, e.g., has been successfully established using the cerebellum in Alzheimer’s disease [39]. However, unaffected brain regions cannot be assumed in PACNS considering its potential global nature. Without pharmacokinetic modeling, globally elevated TSPO expression could be masked, and unspecific uptake mechanism cannot be excluded to contribute to the observed patterns. However, we addressed these concerns by correlating [^18^F]DPA-714 uptake with positive TSPO staining of biopsy samples in two patients as an indicator for specificity. (5) [^18^F]DPA-714 binding is supposed to depend on the Thr147Ala polymorphism. Genotype sequencing of our patients revealed high and intermediate affinity binding types, thus binding genotype should not be an important confounder.

## Conclusion

In this case series, uptake patterns of [^18^F]DPA-714-PET could be correlated to histologically proven PACNS-specific patterns of TSPO expressing perivascular infiltrates. The small number of examined patients limit the conclusions, especially regarding the specificity of the different [^18^F]DPA-714 uptake patterns as well as the sensitivity in the context of early onset immunosuppressive therapy. However, our findings demonstrate the ability of TSPO-PET to visualize the extent as well as hotspots of cerebral inflammation and anti-inflammatory treatment response in patients with PACNS.

## Electronic supplementary material

Suppl. Fig. 1 Flair, Flair/PET overlay and PET of patient #3-#5 (a-i respectively) (PNG 800 kb)

High resolution image (TIF 10587 kb)

Suppl. Fig. 2 Flair, Flair/PET overlay and PET of patient #6-#9 (a-l respectively) (PNG 969 kb)

High resolution image (TIF 14302 kb)

Suppl. Fig 3 (a) TSPO staining demonstrate intermingled microglial cells with TSPO expression as compared to healthy brain tissue of another patient (b) (PNG 691 kb)

High resolution image (TIF 3876 kb)
