# Supplementary figures and images for: Initial experience with [18F]DPA-714 TSPO-PET to image inflammation in primary angiitis of the central nervous system
Source: Eur J Nucl Med Mol Imaging. 2020 Jan 20;47(9):2131–41. doi: 10.1007/s00259-019-04662-4 (PMC7338821; doi:10.1007/s00259-019-04662-4)

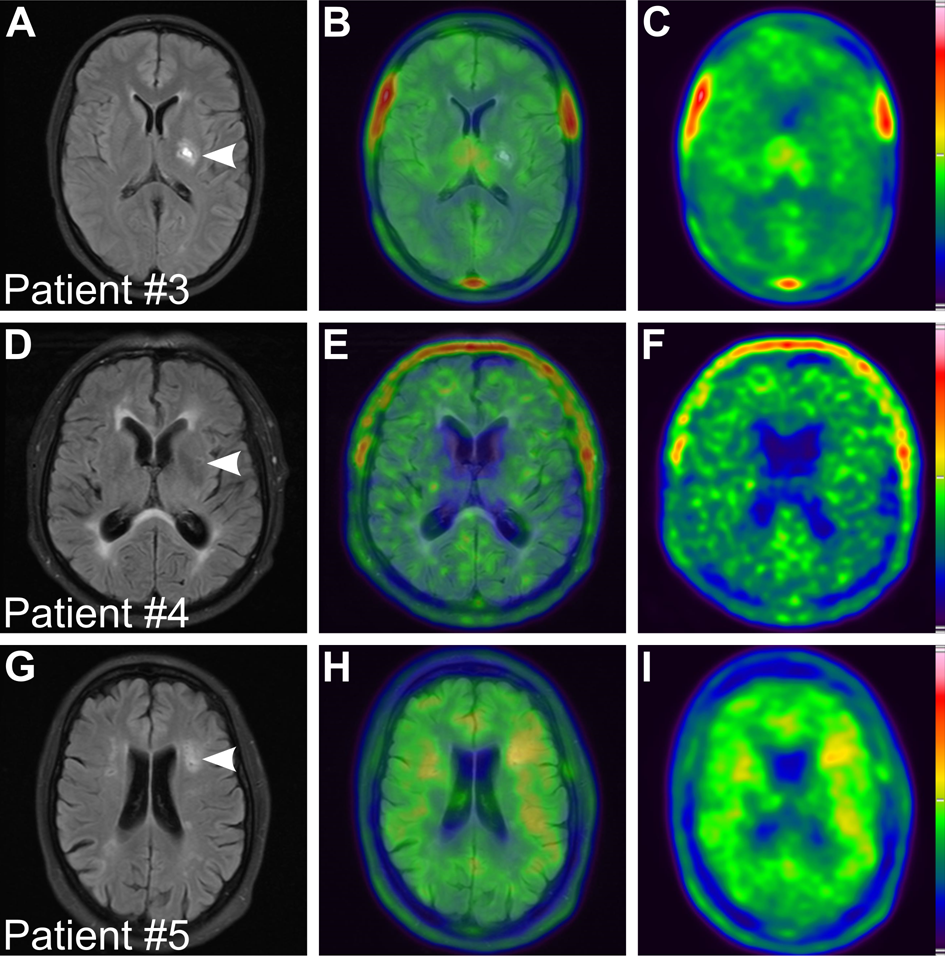

Supplement: Supplementary file 1 — Flair, Flair/PET overlay and PET of patient #3-#5 (a-i respectively) (PNG 800 kb) [file 259_2019_4662_Fig5_ESM.png]

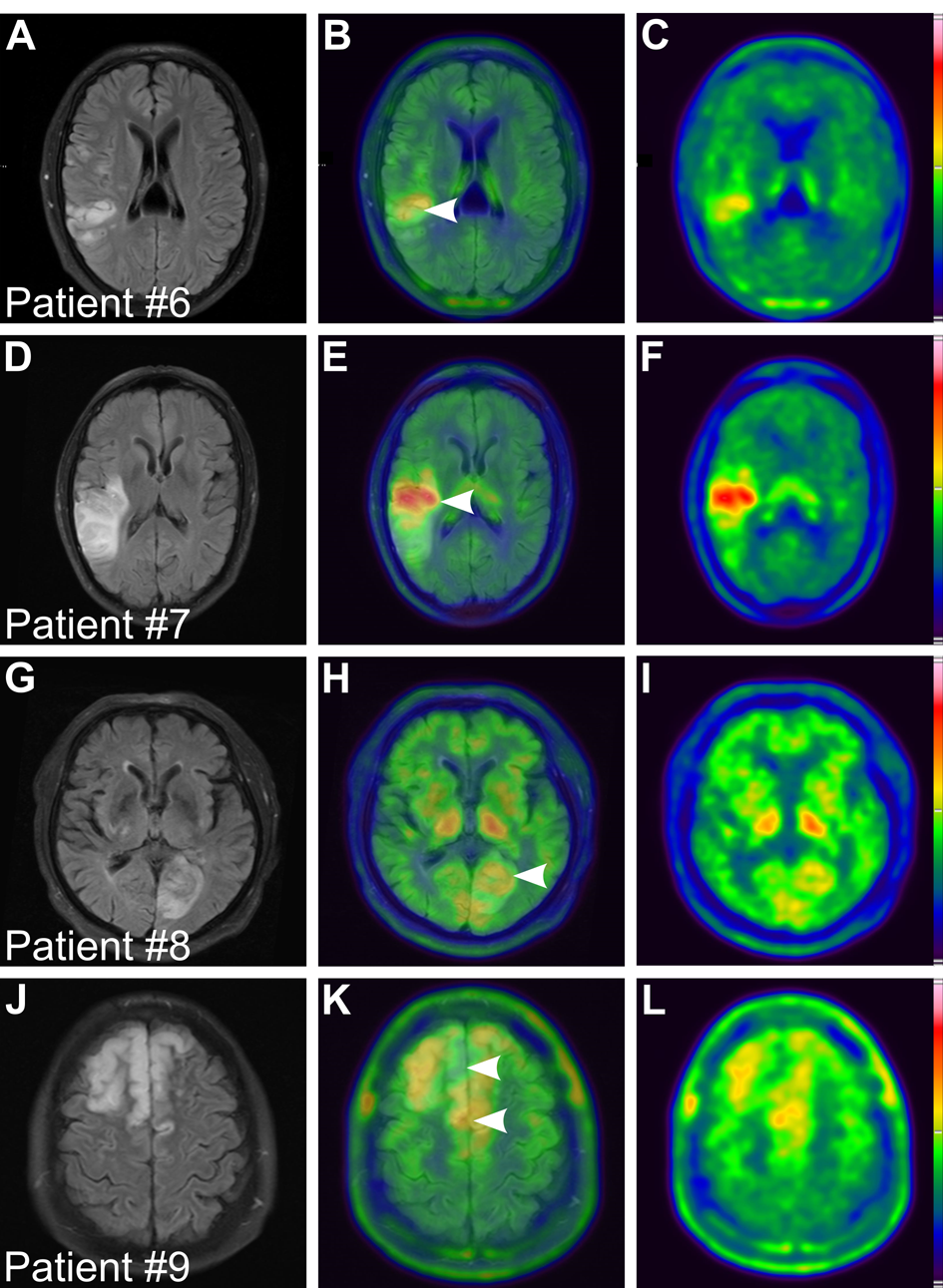

Supplement: Supplementary file 3 — Flair, Flair/PET overlay and PET of patient #6-#9 (a-l respectively) (PNG 969 kb) [file 259_2019_4662_Fig6_ESM.png]

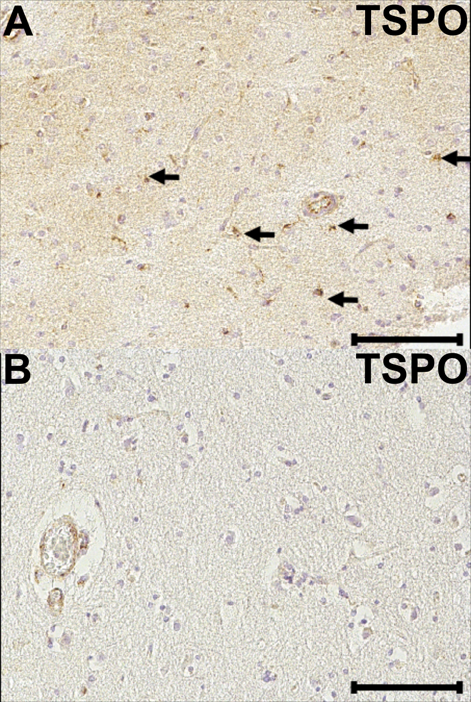

Supplement: Supplementary file 5 — (a) TSPO staining demonstrate intermingled microglial cells with TSPO expression as compared to healthy brain tissue of another patient (b) (PNG 691 kb) [file 259_2019_4662_Fig7_ESM.png]
